# Publication trends in telemedicine research originating from Canada

**DOI:** 10.1177/08404704211070240

**Published:** 2022-01-27

**Authors:** Jim S. Xie, Keean Nanji, Mohammad Khan, Muhammad F. Khalid, Sunir J. Garg, Lehana Thabane, Sobha Sivaprasad, Varun Chaudhary

**Affiliations:** 1 3710 McMaster University, Hamilton, Ontario, Canada.; 2 3710 McMaster University, Hamilton, Ontario, Canada.; 3 150323 Thomas Jefferson University, Philadelphia, PA, USA.; 4 St. Joseph’s Healthcare Hamilton, Hamilton, Ontario, Canada.; 5 3710 McMaster University, Hamilton, Ontario, Canada.; 6 552305 Moorfields Eye Hospital, London, UK.

## Abstract

Telemedicine modalities for patient care have seen significant global uptake during the COVID-19 pandemic. This study aimed to bibliometrically evaluate the evolution and current landscape of telemedicine literature in Canada. The Scopus database was searched to identify telemedicine publications for which the first or last author had a Canadian institutional affiliation. Study selection and data abstraction were conducted by two pairs of independent reviewers. Between 1976 and January 2021, 810 of 3,620 retrieved citations were telemedicine publications originating from Canada, including 29 randomized controlled trials and 6 systematic reviews. The annual publication output increased substantially from 1/year in 1976 to 80/year in 2020. Based on author keyword analysis, the most frequently investigated disciplines or disease entities were primary care, COVID-19, telepsychiatry, heart failure, and mental health. The insights this study provides will aid scientists, policy makers, and other stakeholders in identifying opportunities for future investigation and clinical application.

## Introduction

COVID-19 is an ongoing public health emergency of international concern. The disease is caused by Severe Acute Respiratory Syndrome Coronavirus 2 (SARS-CoV-2), a betacoronavirus that is primarily transmitted through respiratory particles.^1^ Asymptomatic and presymptomatic people are infectious,^2,3^ presenting a major challenge to curtailing spread. Strategies of reducing person-to-person contact, such as travel restrictions, stay-at-home directives, and social distancing mandates, have been implemented on a global scale to reduce transmission. Nonetheless, the rapid spread and high virulence of COVID-19 continue to place significant burden on Healthcare Providers (HCPs) and systems, limiting access to and quality of care.

Telemedicine has emerged as an effective and affordable solution to optimizing care provision while minimizing person-to-person exposure.^4^ The World Health Organization (WHO) defines telemedicine as “the delivery of healthcare services, where distance is a critical factor, by all healthcare professionals using information and communication technologies for the exchange of valid information for diagnosis, treatment, and prevention of diseases and injuries, research and evaluation, and for the continuing education of healthcare providers, all in the interests of advancing the health of individuals and their communities.”^5^ Telemedicine may be live (eg, videoconferencing) or asynchronous (eg, on-line patient portals) and may occur between HCPs and patients or other HCPs.^6^

During the COVID-19 pandemic, virtual care modalities have been used across several medical disciplines and in every stage of care: triage, monitoring and assessment, consultation, treatment, follow-up, and on-line health services.^4,7-10^ Increased uptake of telemedicine has facilitated continuous care to communities and decreased morbidity and mortality related to COVID-19.^4,7^ With the endorsement of major medical organizations worldwide, telemedicine will likely remain an integral component of healthcare post-pandemic.^5,11^ Permanent integration of telemedicine will give rise to numerous challenges that require innovative solutions and further research. In this context, understanding the evolutionary trajectory, current landscape, and key areas of strengths of telemedicine literature may allow stakeholders around the world to develop national strategies to fund and support future telemedicine research.

To date, no comprehensive reviews have examined the evolution of telemedicine research in Canada. Systematic investigations have been conducted to evaluate telemedicine publication trends in other countries.^12,13^ International progress in telemedicine research has also been examined extensively with bibliometric analyses.^14-21^ These studies used broad, search queries for telemedicine without implementing geographical specifications or formal screening of articles.^14-21^ Canada was consistently ranked among the top five countries with the highest telemedicine publication output, demonstrating that Canada remains a leader in telemedicine research.^13-15,19^

Mapping publication trends in telemedicine literature originating from Canada is vital for researchers, institutions, funding bodies, and policy stakeholders to evaluate research areas and clinical settings that require further academic and infrastructural investment. Since the rapid growth in virtual care infrastructure is likely to continue on a global scale post-pandemic, it is currently an opportune time to evaluate the evolution of telemedicine research. This study aims to comprehensively assess trends in Canadian telemedicine research activity between 1976 and January 2021 using a systematic search strategy and bibliometric analysis.

## Methods

The research question was designed according to the PICOT framework: Population: research in telemedicine in Canada; intervention: publication rate; comparator: none; outcome: publications indexed in Scopus; and time: 1976 to January 2021.

### Search strategy

The Scopus scientific citation indexing service (Elsevier, Amsterdam, Netherlands) was searched from its inception up to and including January 2021 for telemedicine studies conducted in Canada. Scopus is the largest abstract and indexing database of peer-reviewed literature. The database contains more than 1.7 billion cited references dating back to 1970 and covers over 7,000 publishers and over 16 million author profiles. Web of Science (Clarivate Analytics, Philadelphia, PA, USA) is a similar international, multidisciplinary indexing database that permits in-depth citation analysis.^22,23^ Ultimately, Scopus was chosen for this study because it covers a wider range of journals.^22,23^ The beginning of the study period was determined by the year in which the earliest Scopus-indexed telemedicine record from Canada was published.

The search strategy was designed in consultation with a research librarian and involved keywords and Medical Subject Headings terms mapping to two concepts: (1) telemedicine and equivalents and (2) Canada and equivalents.

### Selection criteria and data extraction

To ensure that all telemedicine publications could be identified, no restriction was placed on Scopus source type (ie, journal, book, book series, conference proceeding, report, and trade publication). The following document types were included: “article,” “article in press,” “book,” “business article,” “book chapter,” “conference paper,” “conference review,” “editorial,” “letter,” “note,” “press release,” “review,” and “short survey.” Citations of the document-type “erratum” were excluded because they were not considered to represent research productivity.

This paper adopted the World Health Organization’s definition of telemedicine.^5^ Only modalities that involved direct information exchange between healthcare professionals or between healthcare professionals and patients across a distance were considered telemedicine. Accordingly, modalities such as self-management web programs, web sites, and mobile phone applications were considered components of telehealth rather than telemedicine.

Studies were eligible for inclusion if they originated from Canada and had at least one research objective involving telemedicine. Studies were considered to originate from Canada if any of the institutional affiliations of the first and last authors were based in Canada. The corresponding author was presumed to be either the first or last author. Publications were excluded if the full text was not available in English nor French.

Given the bibliometric nature of the study, all references underwent an integrated title, abstract, full-text screening stage. Screening was conducted by two pairs of independent reviewers using Covidence, an on-line systematic review software. A citation was included if both independent reviewers within a pair agreed that all criteria were met. If the title or abstract assessment of either reviewer was uncertain, the full text of the citation was screened using the same eligibility criteria. Disagreements between a pair of reviewers were resolved with arbitration by the other pair of reviewers.

The following data pertaining to all retrieved citations were extracted automatically from Scopus: document type, year of publication, name of the journal in which the article was published if applicable, institutional affiliations of all authors, author keywords, and index keywords. For included articles, two pairs of independent reviewers manually extracted the following data using a standardized, pilot-tested extraction sheet designed in Microsoft Excel for Mac (version 16.49, 2021): (1) whether publications indexed as “article,” “article in press,” or “conference paper” were Randomized Controlled Trials (RCTs); (2) whether publications indexed as “review” or “conference review” were systematic reviews; (3) Journal Impact Factor (JIF) of the source journal based on Journal Citation Reports 2019 if applicable; and (4) CiteScore of the source journal based on Scopus CiteScore 2019 if applicable. Discrepancies between a pair of reviewers were resolved with arbitration by the other pair of reviewers.

### Data analysis

Data were synthesized using the Bibliometrix package (version 3.1.1, 2021) in R (version 4.0.1, 2021). Included publications were imported into R to create a bibliometric data object which was then evaluated for the following endpoints: (1) number of publications per year; (2) number of publications per author, further divided into number of publications per first author and last author; (3) number of publications per journal; (4) frequency with which unique affiliated institutions were cited; (5) number of publications per document type; and 6) frequency with which author keywords (ie, keywords chosen by authors) and index keywords (ie, keywords chosen by Scopus) were cited.

Data were extracted from R into an Excel spreadsheet to calculate summary statistics. Data analysis was performed on all included publications regardless of document type. Unless otherwise stated, medians are reported as median (interquartile range).

## Results

**Figure 1.**
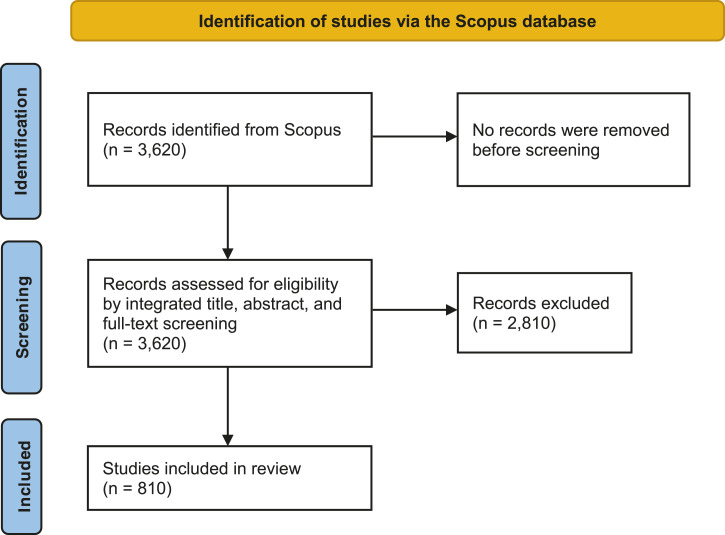
Identification and selection of telemedicine publications originating from Canada based on the search of the Scopus database on February 10, 2021.

A total of 3,620 unique publications from 1976 to January 2021 were retrieved, 810 (22.4%) of which were included because they originated from Canada and pertained to telemedicine (Figure 1). General characteristics of included studies are reported in Table 1. Among the 810 included publications, 610 (75.3%) were indexed as articles, and the remaining publications were conference papers (8.8%), reviews (6.9%), notes (3.5%), editorials (2.1%), letters (1.2%), book chapters (1.2%), short surveys (.6%), conference reviews (.2%), and unlabelled (.1%). Most studies were only available in English (96.9%) and a minority were available in French (2.2%) or both English and French (.9%). Notably, there were only 29 RCTs (3.6%) and 6 systematic reviews (.7%).

**Table 1.** General characteristics of 810 included studies.

Document type, n (%)	
Article	610 (75.3)
Conference paper	71 (8.8)
Review	56 (6.9)
Note	28 (3.5)
Editorial	17 (2.1)
Letters	10 (1.2)
Book chapter	10 (1.2)
Short survey	5 (.6)
Conference review	2 (.2)
Unlabelled	1 (.1)
Study design, n (%)	
Randomized controlled trial	29 (3.6)
Systematic review	6 (.7)
Other	775 (95.7)
Language of publication, n (%)	
English only	785 (96.9)
French only	18 (2.2)
English and French	7 (.9)

**Figure 2.**
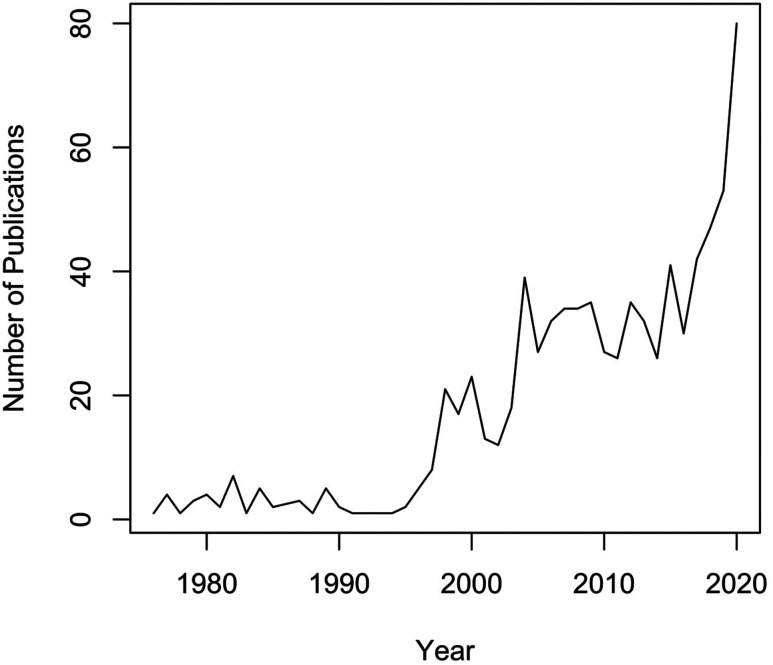
Yearly telemedicine publication output from Canada between 1976 and 2020 inclusive based on the search of the Scopus database on February 10, 2021.

The yearly distribution of included publications is shown in Figure 2. Excluding January 2021, the median number of publications per year was 12.5 (2-32). The median year-on-year growth in publication count was 6.3% (−26.8% to 60.0%) across the entire study period.

The number of individuals authoring Canadian telemedicine studies was 2,746. The median number of publications per author was 1 (1-1). The vast majority of authors (82.3%, n = 2,260) participated in only one publication; only 6.5% (n = 179) of authors had three or more publications. Analyses restricted to the 1,161 unique first and/or last authors in the 810-paper dataset demonstrated similar findings. The median number of publications per first and/or last author was 1 (1-1), and only 6.2% (n = 72) had three or more publications.

The cohort of telemedicine studies originating from Canada included a total of 425 unique institutional affiliations and 3,020 affiliation mentions. The median number of mentions per institution was 1 (1-3). The top 20 most prolific institutions are listed in Table 2. Each of these institutions were cited a median of 67.5 (38.0-143.8) times across the 45-year study period. Including affiliated academic and clinical institutions, the top five institutions were the University of Toronto, the University of Ottawa, the University of British Columbia, the University of Calgary, and the University of Alberta. The top 20 institutions accounted for 68.8% (n = 2,077) of the total number of cited institutional affiliations.

**Table 2.** Top 20 most productive institutions ranked by number of telemedicine research outputs during the period 1976 to 2020 inclusive. For institutions that were closely affiliated with each other or that were constituent bodies of another organization, publication counts were merged and categorized under the principal organization.

Rank	Institution	City	Total outputs (n)	Total outputs (% of dataset, n = 3020)
1	University of Toronto	Toronto	503	16.66
2	University of Ottawa	Ottawa	248	8.21
3	University of British Columbia	Vancouver	185	6.13
4	University of Calgary	Calgary	163	5.40
5	University of Alberta	Edmonton	151	5.00
6	Université Laval	Quebec City	122	4.04
7	Dalhousie University	Halifax	92	3.05
8	Université de Montréal	Montreal	80	2.65
9	Mcmaster University	Hamilton	76	2.52
10	Mcgill University	Montreal	68	2.25
11	University of Manitoba	Winnipeg	67	2.22
12	University of Saskatchewan	Saskatoon	65	2.15
13	Western University	London	58	1.92
14	Queen's University	Kingston	49	1.62
15	University of Victoria	Victoria	44	1.46
16	Université de Sherbrooke	Sherbrooke	36	1.19
17	Simon Fraser University	Burnaby	25	.83
18	Laurentian University	Sudbury	16	.53
19	University of Regina	Regina	15	.50
20	Lakehead University	Thunder Bay	14	.46

The journal that published the most telemedicine studies from Canadian first or last authors was the *Journal of Telemedicine and Telecare* (11.6%, n = 94), followed by *Telemedicine and e-Health* (7.5%, n = 61) and the *Canadian Medical Association Journal* (4.2%, n = 34). Table 3 lists the 22 journals with the highest output representing the top 18 places; the next ten most prolific journals were tied for 19^th^ place. The 22 highest output journals accounted for 46.8% (n = 379) of the 810 publications in the dataset. The median JIF, which is the average number of times a study published within the last two years has been cited in the past year, of the top 22 journals was 1.9 (1.6-3.9). The median CiteScore, which is similar to JIF but spans a 3-year citation period, was 2.4 (1.7-4.0). Overall, the 810-paper dataset originated from 336 unique journals.

**Table 3.** Top 22 most productive journals ranked by number of telemedicine research outputs during the period 1976 to 2020 inclusive. The journal impact factor was obtained from Clarivate Analytics Journal Citation Reports 2019 and CiteScore 2019 measures were obtained from Scopus. The symbol “/” indicates that the journal was not indexed in Journal Citation Reports 2019 or Scopus CiteScore 2019.

Rank	Journal	Total outputs (n)	Total outputs (% of dataset, n = 810)	Impact factor (2019)	CiteScore (2019)
1	Journal of Telemedicine and Telecare	94	11.60	2.616	4.9
2	Telemedicine and e-Health	61	7.53	2.385	4.2
3	CMAJ: Canadian Medical Association Journal/Journal De L’association Medicale Canadienne	34	4.20	7.744	4.0
4	Studies In Health Technology and Informatics	32	3.95	/	0.9
5	Canadian Journal of Ophthalmology	19	2.35	1.369	1.6
5	Journal of Medical Internet Research	19	2.35	5.034	3.9
7	Canadian Journal of Cardiology	12	1.48	5.234	7.1
8	BMC Health Services Research	11	1.36	1.987	3
8	Healthcare Quarterly (Toronto Ont.)	11	1.36	/	0.8
10	Canadian Family Physician	10	1.23	3.112	2.1
11	International Journal of Circumpolar Health	9	1.11	1.217	2.1
12	Canadian Association of Radiologists Journal	8	.99	1.726	2
12	Telemedicine Journal	8	.99	/	/
14	Canadian Journal of Neurological Sciences	7	.86	1.714	2.8
14	Rural and Remote Health	7	.86	1.147	1.5
16	Canadian Journal of Public Health	6	.74	1.638	2.1
16	Journal of Cutaneous Medicine and Surgery	6	.74	1.909	2.4
18	Annals of Family Medicine	5	.62	4.686	5.6
18	British Columbia Medical Journal	5	.62	/	0.7
18	Canadian Journal of Surgery	5	.62	1.61	2.4
18	Hospital Quarterly	5	.62	/	/
18	Trials	5	.62	1.883	3

Author keyword analysis demonstrated that out of 1995 keywords, the top five most frequently used were telemedicine (6.5%), telehealth (5.0%), primary care (1.4%), COVID-19 (1.0%), and e-health (1.0%). Based on author keywords, the five most commonly investigated disciplines or diseases were primary care (1.4%), COVID-19 (1.0%), telepsychiatry (.9%), heart failure (.6%), and mental health (.6%). The terms that headed the list of 21,838 index keywords were more generalized: telemedicine (3.7%), Canada (2.9%), human (2.9%), humans (2.4%), and female (2.2%). The results of author and index keyword analyses are presented in Table 4.

**Table 4.** Top 20 most frequently used author keywords and index keywords in telemedicine literature originating from Canada during the period 1976 to 2020 inclusive.

Rank	Author keyword	Frequency (n)	Frequency (% of dataset, n = 1995)	Index keyword	Frequency (n)	Frequency (% of dataset, n = 21,838)
1	Telemedicine	129	6.47	Telemedicine	802	3.67
2	Telehealth	99	4.96	Canada	643	2.94
3	Primary care	28	1.40	Human	629	2.88
4	COVID-19	19	.95	Humans	533	2.44
5	e-Health	19	.95	Female	489	2.24
6	Canada	18	.90	Article	468	2.14
7	Telepsychiatry	17	.85	Male	465	2.13
8	eHealth	14	.70	Adult	342	1.57
9	eConsult	13	.65	Middle aged	273	1.25
10	Heart failure	12	.60	Aged	258	1.18
11	Access to care	11	.55	Ontario	206	.94
12	Mental health	11	.55	Patient satisfaction	167	.76
13	Rural	11	.55	Organization and management	166	.76
14	Virtual care	10	.50	Healthcare delivery	159	.73
15	Electronic consultation	9	.45	Remote consultation	154	.71
16	Rehabilitation	9	.45	Priority journal	151	.69
17	Self-management	9	.45	Teleconsultation	151	.69
18	Wait times	9	.45	Child	125	.57
19	Medical informatics	8	.40	Adolescent	123	.56
20	Patient satisfaction	8	.40	Videoconferencing	123	.56

## Discussion

This study used a novel approach integrating systematic study identification and bibliometric analysis to delineate the most productive institutions and journals as well as the leading research themes of Canadian telemedicine literature over the past 45 years. While previous bibliometric studies have taken a broader and transnational approach, this study comprehensively assessed the evolution of telemedicine research in Canada.

Research productivity grew substantially from 1 publication per year in 1976 to 80 per year in 2020. The high year-on-year growth in publication count between 1976 and 1990 is largely attributable to the low annual publication output, which resulted in small denominators. In contrast, the overall rise in Canadian telemedicine research is likely due to multiple factors, including innovations in electronic communication, broader access to information and communication technology, and acceptance of technology into day-to-day life.^6,19^ Growth in production and citation of telemedicine research has especially increased since 2020, a trend that was catalyzed by COVID-19.^14^ Indeed, COVID-19 was the fourth most frequently used author keyword in this study’s 45-year dataset despite the pandemic being declared in early 2020.

The temporal course of telemedicine research productivity in Canada parallels increasing publication trends in telemedicine worldwide.^14-16,18-21^ Armfield et al. found that annual publication output in telemedicine and telehealth literature remained consistently low until the mid-1990s, after which productivity grew exponentially; the period 1970-1995 was accordingly named the “early epoch.”^20^ This temporal pattern was corroborated by bibliometric studies of telemedicine and telehealth^16,19^ and of telemedicine in isolation.^15,18^ In alignment with global trends in telemedicine research, this study’s Canadian dataset showed an exponential increase in annual publication output beginning in the mid-1990s (Figure 2). Fatehi and Wootton^21^ (2012) analyzed telemedicine and telehealth publications separately, finding that research productivity in telemedicine increased earlier and to a greater degree than that in telehealth. A more recent bibliometric analysis found that both publication and citation output in telemedicine has increased steadily from 2010 to 2019.^14^ No bibliometric studies of telemedicine publication trends during the COVID-19 pandemic have yet been conducted, but a scoping review found that at least 543 articles on telehealth have been published in the first half of 2020 alone.^9^ There is a general consensus that telemedicine will see rapidly increasing implementation and progress during and after the pandemic,^4,9,14^ a prediction supported by Canadian publication trends.

The accelerated growth of telemedicine infrastructure in Canada and around the world provides a unique opportunity for advancing research and clinical applications in the field. This study has demonstrated a particular need for higher-quality evidence on telemedicine interventions. Only 29 RCTs (3.6%) and 6 systematic reviews (.7%) were found in the 810-paper dataset, a paucity to which competing clinical interests (eg, prioritization of integrating telemedicine into day-to-day practice over interventional research) and the dynamic nature of the field may contribute.

Most institutions involved in telemedicine research were universities and affiliated academic (eg, research centres) and medical institutions (eg, public hospitals). The institutional distribution of publications indicates that the majority of telemedicine research in Canada is conducted in large urban and academic centres.

JIF and CiteScore of journals publishing telemedicine literature have not been examined in previous bibliometric analyses.^8-17^ This study found that the median 2019 JIF and CiteScore of the 22 highest output journals were 1.9 (1.6-3.9) and 2.4 (1.7-4.0), respectively. Less than 30% of journals in the Healthcare Sciences and Services category of Journal Citation Reports had a JIF of 2.8 or higher in 2019.^24^ Thus, telemedicine research originating from Canada has a relatively high overall impact in the field. Although citation metrics have been criticized for their lack of correlation to methodological quality and article importance, higher scores are generally indicative of greater dissemination and influence in the scientific community.^25,26^

Based on author keyword analysis, the predominant disciplines and disease entities studied in Canadian telemedicine literature were primary care, COVID-19, telepsychiatry, heart failure, and mental health. It is likely that the keyword “COVID-19” was frequently used to refer to general telemedicine visits during the pandemic, rather than specific visits involving COVID-19 infections. Nevertheless, these findings highlight potential for further telemedicine research in additional medical disciplines. Previous bibliometric analyses have found similar discipline-specific shifts around the turn of the 21^st^ century from traditionally dominant fields of teleradiology and telepathology to specialities such as telestroke, telecardiology, telepsychiatry, teledermatology, and primary care.^14,19,20^ Shifting patterns in telemedicine research and uptake reinforce that telemedicine is a rapidly evolving field with increasingly diverse functions and applications.^19^ This evolution has been catalyzed by the COVID-19 pandemic and is expected to increase even after the pandemic has resolved.^4,9,14^

Given that telemedicine has been established as a clinical mainstay, health leaders and practitioners should seek to optimize implementation of virtual care modalities across institutions in all stages of care. Notably, there is high potential for growth of telemedicine in medical disciplines other than primary care. To support permanent integration of telemedicine, health researchers should seek to provide higher-quality evidence in the form of RCTs and systematic reviews on the efficacy and utility of virtual care modalities.

### Limitations

Since research outputs were identified from a single citation database, analyses may not reflect the full depth and breadth of telemedicine literature. This study may have missed articles published prior to 1976, in non-indexed journals, and by Canadian institutions for which neither the first nor last author had a Canadian affiliation (eg, international collaboration studies). Furthermore, the search strategy did not include French terms, which may have precluded some French-language telemedicine publications from analyses. To mitigate underestimation of telemedicine publications that do not explicitly state telemedicine but belong to the field nonetheless (eg, teleophthalmology), a thorough literature search was conducted with 29 terms mapped to telemedicine and 17 terms mapped to Canada. Retrieved studies were also formally screened based on prespecified eligibility criteria to exclude publications that are unrelated to telemedicine. These strategies minimized the number of missed and erroneously included studies.

Our analyses did not measure intra- or interdisciplinary collaboration, which may be an interesting avenue of future investigation considering that Canada is a global leader in telemedicine research. Furthermore, analyses did not classify articles based on discipline and/or study design beyond the identification of RCTs and systematic reviews. Classifying studies by these systems would provide deeper insight into research themes and evidence gaps.

## Conclusion

Over the past 45 years, telemedicine research activity in Canada has increased in parallel with global publication trends. Telemedicine research and uptake have risen significantly in the past year due to the COVID-19 pandemic. This rapid growth is expected to increase post-pandemic, cementing telemedicine as an integral component of healthcare around the world. The majority of telemedicine research in Canada is conducted by large academic centres and their affiliated institutions. Based on the JIF and CiteScore of the most productive journals, telemedicine research originating from Canada has a relatively high overall impact in the field. A potential avenue of future research is exploring telemedicine in healthcare disciplines other than primary care. High-quality evidence on telemedicine interventions is also needed to ascertain efficacy. By assessing the content and evolution of Canadian telemedicine literature with a systematic, bibliometric approach, this study will aid clinicians, policy makers, and other stakeholders in developing a national strategy for telemedicine research.
